# Depressive Symptoms in a General Population: Associations with Obesity, Inflammation, and Blood Pressure

**DOI:** 10.4061/2011/740957

**Published:** 2011-12-20

**Authors:** Yüksel Doğan, Altan Onat, Hasan Kaya, Erkan Ayhan, Günay Can

**Affiliations:** ^1^Cardiology Department, Bakirköy Sadi Konuk Education and Research Hospital, 34145 Istanbul, Turkey; ^2^Department of Cardiology, Cerrahpaşa Medical Faculty, Istanbul University, Istanbul, Turkey; ^3^Department of Cardiology, Kartal Koşuyolu Hospital, Istanbul, Turkey; ^4^Department of Cardiology, S. Ersek Cardiovascular Surgery Center, Istanbul, Turkey; ^5^Department of Public Health, Cerrahpaşa Medical Faculty, Istanbul University, Turkey

## Abstract

To determine whether obesity, inflammation, or conventional risk factors are related to depressive symptoms (DeprSy) in the general population. Responses to 3 questions served to assess sense of depression. Body mass index (BMI), C-reactive protein (CRP), and other epidemiologic data of participants were available. In 1940, individuals who consulted a psychiatrist in the preceding year, or felt depressed (together DeprSy), 248 were female. Logistic regressions for adjusted associations of BMI with DeprSy were not significant as was serum CRP level. Diabetes and, in men, fasting glucose concentrations were associated with DeprSy. Systolic blood pressure (SBP) was robustly inversely associated with DeprSy in diverse models at ORs of 0.74 (95%CI 0.63; 0.89) independent of confounders, including antihypertensive and lipid-lowering medication. The use of antidepressants could not explain the reduced BP. Women are predisposed to depression with which, not BMI and CRP, but SBP is inversely associated. Anti-inflammatory substances produced in depressed persons might explain the slightly lower BP.

## 1. Introduction

Evidence is available for the association of both obesity and depressive disorder with increased risk of coronary heart disease (CHD) incidence [1], mortality [2], hypertension [3] and diabetes [4]. Obesity is a greater risk factor than depression for metabolic complications and cardiovascular disease [5]. Depression and severity of depressive symptoms (DeprSy) are both important and independent risk factors for the development and progression of cardiovascular disease in the general population, as well as in patients with cardiovascular disease [6]. However, among 1794 participants of the population-based Canadian Nova Scotia Health Survey, increased inflammation explained only a very small proportion of the association between depression and incident CHD [7]. There may be a link between obesity and depression, but whether depression leads to obesity or obesity causes depression is unclear. A study that confirmed that obesity plays a pivotal role in inflammatory processes relevant to cardiovascular risk in women with polycystic ovary syndrome did not support a correlation of depression with chronic low-grade inflammation in this syndrome [8].

Underlying mechanisms such as chronic inflammation have been extensively investigated as candidate pathways that subsequently link obesity and depression in an attempt to explain how each confers vulnerability to the other and subsequently elevate as the risk for physical illness [9]. A number of cytokines were elevated among depressed individuals, including interleukin-6 (IL-6) and C-reactive protein (CRP) which has been shown to predict cardiac morbidity and mortality [10]. A recent meta-analysis [11], focused on 8 cytokines examined in various studies on depression, found that only TNF-*α* and IL-6 were significantly elevated in depressed compared with control subjects; authors concluded that evidence was strengthened that depression is accompanied by the activation of the inflammatory response system. Little is known about the behavioral and biological mechanisms through which depression becomes associated with inflammation. Some studies suggested that depressive mood may be partly responsible for inflammatory processes, and inflammatory processes may induce depressive symptoms in men [12].

We aimed to investigate whether obesity, systemic inflammation, or a conventional cardiovascular risk factor is related to DeprSy in a representative sample of a middle-aged and elderly general population by using body mass index (BMI), serum CRP as markers, and other risk factors including blood pressure. Such information could serve at least a twofold purpose, namely, (a) regarding utility for screening depressed individuals in the population at large and (b) as a reference information of possible use in future trials with weight-reducing drugs in differentiating potential side effects of depression.

## 2. Methods

### 2.1. Sample Population

This study sample was composed of participants of the 2008/09 follow-up survey of the Turkish Adult Risk Factor (TARF) Study. The TARF is a longitudinal study on the prevalence of cardiac disease and risk factors in a representative sample of adults in Turkey carried out periodically almost biennially since 1990 in 59 communities throughout all geographical regions of the country [13]. Details of the overall sampling were described previously [14]. The study was approved by the Ethics Committee of the Istanbul University Medical Faculty. Written informed consent was obtained from all participants. Partial logistic support was provided by the Turkish Ministry of Health. Data were obtained by history of the past years via a structured questionnaire, physical examination of the cardiovascular system, sampling of blood, and recording of a resting 12-lead electrocardiogram. The study group which consisted of 3/5 s of the whole cohort was similar to the latter with respect to age, sex distribution, proportions of MetS, and type-2 diabetes.

### 2.2. Assessment of Depressive Mood by Interview

Following 3 questions were directed to participants during face-to-face interview by a physician at his 4th year of specialization. Questions were to be replied by Yes/No; the second query included a subsidiary question. Clarification was given by interviewer, when necessary.

1. Did you consult in the past year a physician due to a change in your mood or spirits?
2. Do you feel depressed? If so, do you use antidepressant drugs?
3. Have there been periods that life is not worth living or that you thought of seriously harming yourself?

### 2.3. Measurement of Risk Factors

Blood pressure was measured with an aneroid sphygmomanometer (Erka, Bad Tölz, Germany) in the sitting position on the right arm, and the mean of two recordings 5 min apart was recorded. Height was measured without shoes using a measuring stick and weight in light indoor clothes using scales. BMI was computed as weight divided by height squared (kg/m^2^).

*Blood samples* were collected, spun at 1000 g for 10 minutes, and shipped on cooled gel packs at 2–5°C to Istanbul to be stored in deep freeze at −75°C, until analyzed at a central laboratory. Serum concentrations of hsCRP were measured by nephelometry (BN Prospec, Behring Diagnostics, Westwood, MA, USA). Serum concentrations of total cholesterol, fasting triglycerides, glucose, HDL cholesterol (HDL-C plus 2nd generation, directly without precipitation), and LDL cholesterol (directly) were determined by using enzymatic kits from Roche Diagnostics (Mannheim, Germany) with a Hitachi 902 autoanalyzer. Serum fasting concentrations of insulin were carried out by the electrochemiluminescence immunoassay ECLIA on Roche Elecsys 2010 using Roche kits (Roche Diagnostics, Mannheim, Germany).

### 2.4. Definitions

In regard to cigarette smoking, nonsmokers, former smokers, and current smokers formed the categories. Anyone who drank alcoholic drinks once a month or more frequently was considered as alcohol consumer. *Type 2 diabetes *was diagnosed with the criteria of the American Diabetes Association [15], namely, by self-report or when plasma fasting glucose was ≥7 mmol/L or when 2-h postprandial glucose was >11 mmol/L.

### 2.5. Data Analysis

Descriptive parameters were shown as mean ± standard deviation, estimated mean ± standard error, or in percentages. Due to the skewed distribution, values derived from log-transformed (geometric) means were used for CRP and insulin. *t*-tests and Pearson's chi-square tests were used to analyze the differences between means and proportions of groups. Likelihood estimates and 95% confidence intervals (CIs) were obtained by the use of logistic regression analyses in models that adjusted for potential confounders in which the odds ratio (OR) was expressed in terms of 1-SD increment of the independent variable. A value of *P* < 0.05 on the two-tail test was considered statistically significant. Statistical analyses were performed using SPSS-10 for Windows.

## 3. Results

**Figure 1:**
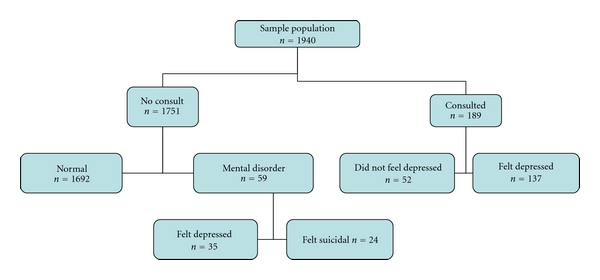
Diagrammatic representation of the responses of participants to the interview.

1940 men and women who were interviewed in the months of September of 2008 and of 2009 formed the sample population; 966 (49.8%) were female, and 974 (50.2%) male. 1692 people (87.2%) negated all questions; these are referred to as “healthy” (Figure 1). The remaining 248 individuals who responded with 1 or more positive answers are referred to as having a depressive symptoms (DeprSy). 189 subjects (9.8%) consulted a physician (referred to as “consulters”), 52 of whom did not feel depressed and 137 felt depressed, (7%) [87 consulters used antidepressants, 25 felt suicidal]. Fifty-nine subjects (3%) did not consult a physician, yet 35 of these felt depressed, [13 used antidepressants and 4 felt suicidal] and 24 subjects felt suicidal.

Females were significantly more often DeprSy than males (19.8% versus 6/2%, *P* < 0.001) (Table 1). Women with DeprSy were by 2.9 years younger and had by 6.4/2.6 mmHg lower BP values than healthy women (*P* = 0.001 in each). Among men, no significant differences were found between these groups. Presence of CHD (*P* = 0.69 in men, 0.95 in women) or diabetes (*P* = 0.12 in men, 0.47 in women) was not related to DeprSy. Prevalence of current smoking was not different among DeprSy in either sex, nor was that of alcohol usage.

### 3.1. Association of Individuals with Depressive Symptoms and Other Variables

Table 2 shows results of a logistic regression analysis for the association of DeprSy. The first model comprised sex, age, BMI, and SBP and disclosed that SBP and sex were significant variables (the odds of DeprSy in women was 4-fold the odds for men). In Model 2, smoking status and fasting glucose, usage of antihypertensive, and lipid-lowering drugs were added to the previous variables. SBP retained its independent significant OR. One SD increment of SBP was inversely associated with DeprSy, at an OR 0.74 in the total sample. This did not reach significance in men but showed a similar OR as in women. Age, smoking status, and BMI persisted not to be associated. Fasting glucose was significantly associated only in men, OR being 1.27 (95%CI 1.00; 1.55), and at borderline significance in combined genders. When diabetes replaced fasting glucose in the latter model (comprising 1771 subjects with 215 DeprSy), OR was borderline significant 1.47 (95%CI 0.99; 2.18) in combined genders but did not reach significance in men alone [1.65 (0.83; 3.28)]. Overweight women, compared with normal-weight women, tended weakly to DeprSy (OR 1.47, *P* < 0.14), after adjustment for age and smoking status.

### 3.2. Stratified Analysis by Use of Antidepressants

When the association between DeprSy and SBP was scrutinized by stratifying for individuals using antidepressive drugs, participants reporting the use of antidepressants (*n* = 100) had an age-adjusted mean SBP only 1.3 mmHg lower in men (*P* = 0.71), 0.9 mmHg lower in women (*P* = 0.73), than the remainder of the study sample.

When only consulters were analyzed by multiple logistic regression again in the 2 similar models (Table 3), excluding those who were in depressed mood but somehow did not consult a physician, fasting glucose was not associated though sex and (inversely) SBP proved to be significantly associated at virtually identical ORs of 0.75.

In a further model, we examined in 1745 subjects the association of CRP with DeprSy, along with BMI, SBP, and other variables (Table 4, model 2). CRP, BMI, and smoking status persisted not to be associated whereas female sex (4-fold), and, inversely, SBP was highly significantly associated [OR 0.72 (95%CI 0.60; 0.85]. Serum CRP disclosed for a 3-fold increment an OR of 0.98 (95%CI 0.88; 1.08).

## 4. Discussion

Salient findings in this population-based study seeking the relationship between depression and obesity/inflammatory markers and conventional cardiovascular risk factors was that, while BMI and serum CRP level were not associated, female sex and fasting glucose were significantly associated with DeprSy. Noteworthy was that SBP was inversely and robustly associated with DeprSy in the total study sample.

Our finding relative to a current depressive disorder in this middle-aged population to prevail in 6.2% in men, and 19.8% of women is coarsely in line with the reported prevalence of a major lifetime depression among 6914 young US adults from the NHANES-III survey as 5.7% in men and 11.7% in women, corresponding to a two-fold risk in women as in men [16]. Among 1600 Chinese people aged 60 years or over, the age-standardized prevalence of depression was found as 6%, and female sex was independently associated [17]. The preponderance of women was stronger among Turks inasmuch as the multivariably adjusted OR of DeprSy for women was consistently 3.9-fold than the odds for men. Age was not related to DeprSy in men but younger age tended to be a predisposing covariate among women. Younger age and female sex correlated significantly with depression also in persons with severe obesity [18]. DeprSy was independently associated with fasting glucose in men and tended to be so with the presence of diabetes in both genders. Though this association was weaker, it is in concordance with a previous meta-analysis in which the presence of diabetes was shown to double the odds of comorbid depression [19].

### 4.1. Lack of Association of BMI and CRP with Depression

Mean BMI in the NHANES-III survey was not significantly different in persons with than without a major depression (25.8 versus 25.4 kg/m^2^, *P* = 0.42) (16) though adjusted analyses were not made.

Among young US adults from the NHANES-III survey, an elevated CRP (≥2.14 mg/L) was observed in 13.7% of men and 27.3% in women. Elevated levels were not associated in multivariably adjusted with current or lifetime depression in females with which our findings are in agreement. No difference in BMI, nor in TNF-*α* or IL-6 concentrations, was observed with depression in 63 obese Polish women [20]. Analysis of extensive inflammatory biomarkers and adiposity measures in 57 patients with polycystic ovary syndrome and 28 healthy women did not support a correlation of depression with chronic low-grade inflammation in women with this syndrome [8]. The case-control study by Miller on 100 young adults [21] also did not support a model in which inflammatory molecules arising from expanded adipose tissue promoted depressive symptoms but rather that depressive symptoms promoted weight accumulation with subsequent low-grade inflammation.

Level of IL-6 was not independently associated with elevated depressive symptoms also in a study on 416 Finns, but levels of the anti-inflammatory IL-1 receptor antagonist were exhibited an OR over 2-fold compared to those not belonging to the group with elevated depressive symptoms [22]. Authors concluded that the pronounced secretion of the anti-inflammatory marker IL-1 receptor antagonist may reflect the presence of compensatory mechanisms during a depression-related inflammatory state.

### 4.2. Inverse Relation of Blood Pressure to Depression

The surprising and main finding in the current study was that BP was lower in depressed subjects (in the order of 5/2 mmHg) which could not be accounted for by age, smoking status, BMI, glucose, or CRP. The odds for DeprSy in multivariable analyses were consistently lower by one-quarter for a 1-SD increment in SBP in each gender (though failing to reach significance in men because of limited statistical power). Although reasons for it may be unclear, this association was robust, exhibiting a narrow confidence interval and preserving the OR in variously adjusted models in each gender. In subgroup analyses, we could exclude the use of antidepressants as possibly accounting for this finding.

This finding does not seem to be specific for the current study. Though in elderly Chinese, not detected hypertension but undetected severe hypertension (≥160/95 mmHg) was reported to be associated with depression (OR 1.78; [95%CI 1.05; 3.01]) (17) that may have been related to a high BMI or to diabetes for which adjustment was not made. In young US adults from the NHANES-III survey, mean SBP was lower by 1.5 mmHg in persons with than without a major depression (*P* = 0.09) [16]. A relationship between depression and low SBP was shown in Koreans in whom the lowest SBP decile of the study sample, regardless of antihypertensive medication, had significantly elevated odds for depression compared to the median quintile of the sample [23]. Moreover, previous studies reported low systolic BP in segments of population in whom self-perceived physical and mental well-being was low [24, 25]. Finally, through the examination of the National Nursing Home Survey database, Rosengren et al. [26] found that the use of any antihypertensive agent (among over 7200 nursing home residents) was associated with a decreased likelihood (0.76 [95%CI 0.63; 0.90]) of a diagnosis of depression.

It has been proposed that anti-inflammatory substances (including anti-inflammatory cytokines IL-4 and IL-10) or IL-1 receptor antagonist [22] may compensate for pronounced proinflammatory changes in depressed individuals. The latter is a specific inhibitor of the depression-related cytokines IL-1*α* and IL-1*β* secreted by activated monocyte/macrophage lineage cells [27]. The development of prehypertension among Turks, especially women, has been shown by us to be significantly predicted by pro-inflammatory status marked by CRP [28] and/or apoA-I levels (unpublished observations). It may be hypothesized that anti-inflammatory substances, produced in depressed persons, might counteract pro-inflammatory state and result in slightly lower BP than in nondepressed individuals in whom this response may be less active. In fact, a concept referring to an adaptive response to the pronounced secretion of inflammatory substances has been designated as compensatory anti-inflammatory response syndrome [29].

**Limitations**

The assessment of depression herein is limited by using the indicators of such a disorder rather than a diagnosed depression. The reliability and validity of our questionnaire has not been formally tested in community samples, but it is congruent with the essence of the Beck Depression Inventory commonly utilized in clinical [18] or epidemiologic studies. Responses to treatment and treatment seeking might not perfectly correspond to DeprSy or depression. The cross-sectional design precludes the drawing of conclusions on causality of present findings. Though an association between CRP and depressive disorder was lacking, a possible association of inflammation cannot be excluded in this sample, considering that association with depression of inflammatory mediators other than CRP (chemokines and cytokines such as MCP-1, MIP-1*α*, TNF*α*, IL-1*β*, IL-6, interferon activity, etc. [30]) originating from tissues other than adipose tissue such as macrophages and T cells [11] has been demonstrated. Main findings cannot be attributed to a type-2 error, given both the robust result under diverse regression models and their consistency with available findings elsewhere, albeit scarce. Additional sensitivity analysis for depression treatment failed to show a significant association. The current population-based study on a large cohort in which measurements for anthropometric and lipid and nonlipid variable were available for performing multivariable adjustments form the strength of this study.

We *conclude* that, in a middle-aged and elderly general population, a depressive symptoms prevail at 4-fold odds in women. BMI and CRP were not associated, but fasting glucose was associated in men with likelihood for DeprSy. Surprisingly, systolic BP was robustly associated inversely with depression which may be attributable to a compensatory anti-inflammatory response in depressed persons, reversing a tendency to prehypertension mediated by pro-inflammatory state in this population. This observation needs confirmation in other populations.

## Figures and Tables

**Table 1:** Baseline characteristics of the sample population (*n* = 1940), by gender and mental disorder.

	Women (*n* = 966)				Men (*n* = 974)			
	Healthy (*n* = 778)		Depres. disorder* (*n* = 188)		Healthy (*n* = 914)		Depres. disorder* (*n* = 60)	
	Mean	SD	Mean	SD	Mean	SD	Mean	SD
Age, years	57.5	11	**54.6**	9.8	56.4	11	56.3	11.7
Systolic blood pressure, mmHg	129.0	21	**122.6**	21.3	121.9	19	118.6	16.4
Diastolic blood pressure, mmHg	77.0	10.3	**74.4**	10	74.7	10	74.1	9.6
Current smoker, *n*. %	92	13	26	15.4	306	38	18	36
Alcohol user, *n*. %	2	0.3	2	1.2	102	12.5	7	13.7
Body mass index, kg/m^2^	31.2	6	31.3	5.6	28.2	4.3	27.8	4.1
Fasting glucose, mg/dL	102.3	40.2	104.8	48	102.7	39.7	116.1	60.6
Total cholesterol, mg/dL	199.7	44.4	*206*	42.1	198	41.8	192.6	37.7
HDL cholesterol, mg/dL	48.1	12.4	49.6	13.5	40.6	11	40.9	8
Fast. triglycerides, mg/dL	177.2	117	190.7	168	177.2	117	190.7	168
Fasting insulin, mIU/L	8.64	2.02	8.74	2.00	8.28	2.05	9.17	2.04
C-reactive protein, mg/L	2.50	2.88	2.35	2.92	2.04	2.73	1.73	2.9
Presence of CHD, *n*. %	80	10.3	19	10.2	122	13.4	9	15.3
Presence of diabetes, *n*. %	119	15.5	33	17.6	135	15.3	13	23.2

*includes those who consulted a physician or felt depressed without having consulted a physician.

*P* < 0.002 in boldface, 0.067 in italics; all other values between healthy and mental disorder are >0.1.

**Table 2:** Logistic regression analysis for the association of certain parameters with depressive disorder, by gender.

	*Total*		*Men*		*Women*	
	OR 95% CI		OR 95% CI		OR 95% CI	
*Model 1*	241/1919^†^		57/963^†^		184/956^†^	

Female gender	**3.97**	2.87; 5.49
Age, 11 years	0.91	0.77; 1.06	1.07	0.82; 1.41	*0.85*	0.70; 1.02
Body mass index, 5 kg/m^2^	1.08	0.94; 1.23	0.97	0.69; 1.35	1.10	0.96; 1.28
Systolic b. pressure, 20 mmHg	**0.74**	0.63; 0.87	0.80	0.58; 1.10	**0.72**	0.60; 0.89

*Model 2*	189/1594^†^		49/822^†^		140/772^†^	

Female gender	**3.48**	2.20; 5.52
Age, 11 years	0.90	0.76; 1.07	1.04	0.77; 1.44	0.84	0.68; 1.03
Body mass index, 5 kg/m^2^	1.01	0.87; 1.18	0.99	0.85; 1.43	1.03	0.87; 1.22
Systolic b. pressure, 20 mmHg	**0.74**	0.62; 0.89	0.75	0.52; 1.06	**0.74**	0.60; 0.92
Fasting glucose, 40 mg/dL	*1.13*	1.00; 1.32	**1.27**	1.00; 1.55	1.08	0.89; 1.27
Current versus never smoking	0.83	0.45; 1.53	0.92	0.42; 2.00	0.99	0.30; 3.28
Antihypert. drug use, yes/no	1.19	0.83; 1.73	1.20	0.73; 1.81	1.17	0.76; 1.79
Lipid-lower. drug use, yes/no	1.08	0.63; 1.88	1.07	0.54; 2.08	1.03	0.54; 1.97

^†^number of depressive disorder/total number.

201/61 antihypertensive/lipid-lowering drug usage in men, 322/75 usage in women, respectively.

Significant values are highlighted in boldface.

**Table 3:** Logistic regression analysis for the association of certain parameters with individuals consulting a physician, by gender.

	*Total*		*Men*		*Women*	
	OR 95% CI		OR 95% CI		OR 95% CI	
*Model 1*	184/1919^†^		42/963^†^		142/956^†^	

Female gender	**3.98**	2.76; 5.76
Age, 11 years	0.95	0.79; 1.12	1.18	0.88; 1.61	0.86	0.70; 1.06
Body mass index, 5 kg/m^2^	1.07	0.93; 1.24	0.92	0.62; 1.36	1.10	0.94; 1.29
Systolic b. pressure, 20 mmHg	**0.75**	0.63; 0.92	0.80	0.56; 1.17	**0.75**	0.62; 0.94

*Model 2*	147/1594^†^		36/822^†^		111/772^†^	

Female gender	**3.39**	2.02; 5.68
Age, 11 years	0.91	0.75; 1.10	1.09	0.76; 1.56	0.84	0.66; 1.06
Body mass index, 5 kg/m^2^	1.05	0.89; 1.23	0.94	0.61; 1.46	1.07	0.89; 1.28
Systolic b. pressure, 20 mmHg	**0.75**	0.62; 0.94	0.75	0.51; 1.13	*0.79*	0.62; 1.00
Fasting glucose, 40 mg/dL	0.96	0.82; 1.17	1.08	0.79; 1.43	0.92	0.76; 1.17
Current versus never smoking	0.80	0.40; 1.60	0.97	0.40; 2.35	0.95	0.25; 3.59
Antihypert. drug use, yes/no	1.23	0.82; 1.85	*1.99*	0.91; 4.34	1.05	0.65; 1.68
Lipid-lower. drug use, yes/no	0.92	0.48; 1.75	0.90	0.26; 3.15	0.90	0.43; 1.90

^†^number of consulters/total number.

Antihypertensive/lipid-lowering drug usage in 523/136 men and women, respectively.

Significant values are highlighted in boldface.

## References

[1] Ferketich AK, Schwartzbaum JA, Frid DJ, Moeschberger ML (2000). Depression as an antecedent to heart disease among women and men in the NHANES I study. *Archives of Internal Medicine*.

[2] Schulz R, Beach SR, Ives DG, Martire LM, Ariyo AA, Kop WJ (2000). Association between depression and mortality in older adults: the Cardiovascular Health study. *Archives of Internal Medicine*.

[3] Davidson K, Jonas BS, Dixon KE, Markovitz JH (2000). Do depression symptoms predict early hypertension incidence in young adults in the CARDIA study? Coronary artery risk development in young adults. *Archives of Internal Medicine*.

[4] Nichols GA, Brown JB (2003). Unadjusted and adjusted prevalence of diagnosed depression in type 2 diabetes. *Diabetes Care*.

[5] Rexrode KM, Buring JE, Manson JE (2001). Abdominal and total adiposity and risk of coronary heart disease in men. *International Journal of Obesity*.

[6] Miller GE, Stetler CA, Carney RM, Freedland KE, Banks WA (2002). Clinical depression and inflammatory risk markers for coronary heart disease. *American Journal of Cardiology*.

[7] Davidson KW, Schwartz JE, Kirkland SA (2009). Relation of inflammation to depression and incident coronary heart disease (from the Canadian Nova Scotia Health Survey [NSHS95] Prospective Population Study). *American Journal of Cardiology*.

[8] Benson S, Janssen OE, Hahn S (2008). Obesity, depression, and chronic low-grade inflammation in women with polycystic ovary syndrome. *Brain, Behavior, and Immunity*.

[9] Goldstein BI, Kemp DE, Soczynska JK, McIntyre RS (2009). Inflammation and the phenomenology, pathophysiology, comorbidity, and treatment of bipolar disorder: a systematic review of the literature. *Journal of Clinical Psychiatry*.

[10] Danesh J, Collins R, Peto R, Fibrinogen R (2000). C-reactive protein, albumin, and white cell count: meta-analyses of prospective studies of coronary heart disease. *Journal of American Medical Association*.

[11] Dowlati Y, Herrmann N, Swardfager W (2010). A meta-analysis of cytokines in major depression. *Biological Psychiatry*.

[12] Elovainio M, Aalto AM, Kivimäki M (2009). Depression and C-reactive protein: population-based health 2000 study. *Psychosomatic Medicine*.

[13] Onat A, Avcı GŞ, Şenocak M, Örnek E, Gözükara Y (1992). Plasma lipids and their interrelation in Turkish adults. *Journal of Epidemiology, Community Health*.

[14] Onat A (2001). Risk factors and cardiovascular disease in Turkey. *Atherosclerosis*.

[15] American Diabetes Association (2008). Diagnosis and classification of diabetes mellitus. *Diabetes Care*.

[16] Ford DE, Erlinger TP (2004). Depression and C-reactive protein in US adults: data from the third national health and nutrition examination survey. *Archives of Internal Medicine*.

[17] Chen R, Wei L, Hu Z, Qin X, Copeland JRM, Hemingway H (2005). Depression in older people in rural China. *Archives of Internal Medicine*.

[18] Dixon JB, Dixon ME, O’Brien PE (2003). Depression in association with severe obesity: changes with weight loss. *Archives of Internal Medicine*.

[19] Anderson RJ, Freedland KE, Clouse RE, Lustman PJ (2001). The prevalence of comorbid depression in adults with diabetes: a meta-analysis. *Diabetes Care*.

[20] Olszanecka-Glinianowicz M, Zahorska-Markiewicz B, Koceak P (2009). Is chronic inflammation a possible cause of obesity-related depression?. *Mediators of Inflammation*.

[21] Miller GE, Freedland KE, Carney RM, Stetler CA, Banks WA (2003). Pathways linking depression, adiposity, and inflammatory markers in healthy young adults. *Brain, Behavior, and Immunity*.

[22] Lehto SM, Niskanen L, Miettola J, Tolmunen T, Viinamäki H, Mäntyselkä P (2010). Serum anti-inflammatory markers in general population subjects with elevated depressive symptoms. *Neuroscience Letters*.

[23] Kim BS, Bae JN, Cho MJ (2010). Depressive symptoms in elderly adults with hypotension: different associations with positive and negative affect. *Journal of Affective Disorders*.

[24] Simon NM, McNamara K, Chow CW (2008). A detailed examination of cytokine abnormalities in major depressive disorder. *European Neuropsychopharmacology*.

[25] Simonson W, Han LF, Davidson HE (2011). Hypertension treatment and outcomes in US nursing homes: results from the US national nursing home survey. *Journal of the American Medical Directors Association*.

[26] Rosengren A, Tibblin G, Wilhelmsen L (1993). Low systolic blood pressure and self perceived wellbeing in middle aged men. *British Medical Journal*.

[27] Dinarello CA (2009). Immunological and inflammatory functions of the interleukin-1 family. *Annual Review of Immunology*.

[28] Onat A, Yazici M, Can G, Kaya Z, Bulur S, Hergenç G (2008). Predictive value of prehypertension for metabolic syndrome, diabetes, and coronary heart disease among Turks. *American Journal of Hypertension*.

[29] Adib-Conquy M, Cavaillon JM (2009). Compensatory anti-inflammatory response syndrome. *Thrombosis and Haemostasis*.

[30] Wessely S, Nickson J, Cox B (1990). Symptoms of low blood pressure: a population study. *British Medical Journal*.

